# Do support groups members disclose less to their partners? The dynamics of HIV disclosure in four African countries

**DOI:** 10.1186/1471-2458-13-589

**Published:** 2013-06-17

**Authors:** Anita Hardon, Gabriela B Gomez, Eva Vernooij, Alice Desclaux, Rhoda K Wanyenze, Odette Ky-Zerbo, Emmy Kageha, Ireen Namakhoma, John Kinsman, Clare Spronk, Edgar Meij, Melissa Neuman, Carla Makhlouf Obermeyer

**Affiliations:** 1 Amsterdam Institute for Social Science Research, University of Amsterdam, Kloveniersburgwal 48, 1012 CX, Amsterdam, The Netherlands; 2 Department of Global Health, Academic Medical Centre, University of Amsterdam and Amsterdam Institute for Global Health and Development, Pietersbergweg 17, PO Box 22700, 1100 DE, Amsterdam, The Netherlands; 3 Institut de Recherche pour le Développement, TRANSVIHMI, Dakar, Senegal; 4 Makerere University School of Public Health, New Mulago Hospital Complex, PO Box 7072, Kampala, Uganda; 5 Programme d’Appui au Monde Associatif & Communautaire de Lutte Contre le VIH/SIDA (PAMAC), 11 BP 1023 CMS 11, Ouagadougou, Burkina Faso; 6 Research for Equity and Community Health (REACH) Trust, Paul Kagame Highway, 6/28, PO Box 1597, Lilongwe, Malawi; 7 Umeå Centre for Global Health Research, Umeå University, Umeå, SE-901 85, Sweden; 8 Intelligent Systems Lab Amsterdam, University of Amsterdam, Science Park 904, 1098 XH, Amsterdam, The Netherlands; 9 Institute for Global Health, University College London, Guilford Street 30, WC1N 1EH, London, UK; 10 Faculty of Health Sciences, American University of Beirut, PO Box 11-0236, Riad El-Solh, Beirut, Lebanon

**Keywords:** Disclosure, HIV counselling and testing, Support groups, Stigma, HIV/AIDS, Mixed methods

## Abstract

**Background:**

Recent efforts to curtail the HIV epidemic in Africa have emphasised preventing sexual transmission to partners through antiretroviral therapy. A component of current strategies is disclosure to partners, thus understanding its motivations will help maximise results. This study examines the rates, dynamics and consequences of partner disclosure in Burkina Faso, Kenya, Malawi and Uganda, with special attention to the role of support groups and stigma in disclosure.

**Methods:**

The study employs mixed methods, including a cross-sectional client survey of counselling and testing services, focus groups, and in-depth interviews with HIV-positive individuals in stable partnerships in Burkina Faso, Kenya, Malawi and Uganda, recruited at healthcare facilities offering HIV testing.

**Results:**

Rates of disclosure to partners varied between countries (32.7% – 92.7%). The lowest rate was reported in Malawi. Reasons for disclosure included preventing the transmission of HIV, the need for care, and upholding the integrity of the relationship. Fear of stigma was an important reason for non-disclosure. Women reported experiencing more negative reactions when disclosing to partners. Disclosure was positively associated with living in urban areas, higher education levels, and being male, while being negatively associated with membership to support groups.

**Conclusions:**

Understanding of reasons for disclosure and recognition of the role of support groups in the process can help improve current prevention efforts, that increasingly focus on treatment as prevention as a way to halt new infections. Support groups can help spread secondary prevention messages, by explaining to their members that antiretroviral treatment has benefits for HIV positive individuals and their partners. Home-based testing can further facilitate partner disclosure, as couples can test together and be counselled jointly.

## Background

The coverage of antiretroviral treatment (ART) [1] in Sub-Saharan Africa has expanded significantly in recent years. New guidelines for eligibility and recent evidence showing the public health benefits of early ART initiation in preventing onward transmission [2-5] have led to a prioritization of testing to diagnose patients at earlier stages of HIV infection. Key issues for treatment as prevention programs include outreach to key populations and disclosure among sero-discordant couples.

Within HIV counselling and testing programs, HIV-positive individuals are generally actively encouraged to disclose to their partners. Despite this advice, disclosure to partners remains sub-optimal in many settings in Sub-Saharan Africa [6,7], with a significant proportion of HIV-positive individuals waiting over a year to inform their partners [8,9]. Rates of disclosure within countries differ by ethnicity, gender and by the kind of testing facility [10-14].

Although studies have found that health facilities offering HIV testing in Sub-Saharan Africa are trying to accommodate the needs of couples, uptake remains a challenge, with a low number of couples testing together [15]. Nevertheless, some studies have reported success in increasing testing among couples [16], in particular through home-based testing [17,18], while one recent study on HIV testing within routine health services [19] reported high rates of partner referral in post-test counselling.

Fear of enacted stigma – including violence, abandonment and divorce – negatively affects rates of partner disclosure [8,15,20-25]. The Multi-Country African Testing and Counselling for HIV (MATCH) study [19] conducted in Burkina Faso, Kenya, Malawi and Uganda found that despite the emphasis on partner disclosure in post-test counselling, only 37% of the HIV-positive women tested within Prevention of Mother-to-Child Transmission (PMTCT) programmes had disclosed to their partners. When they did so, disclosure often led to serious rifts, including abandonment and divorce. Men disclosed more often and experienced less negative consequences upon disclosing than women [26]. Partner disclosure requires trust that the intimate other will provide care and will not contribute to the stigmatization of the affected individual.

Small-scale ethnographic studies suggest that HIV-positive individuals find it easier to disclose to other HIV-positive persons in support groups or in clinics than to their partners. These studies also suggest that HIV-positive individuals often pursue strategies of incremental, partial or indirect disclosure, which in some cases allow their partners to disclose first [27-29].

The current study employs mixed methods to explore the dynamics and consequences of partner disclosure in Burkina Faso, Kenya, Malawi and Uganda. In doing so, it integrates data from the qualitative and quantitative components of the MATCH study [30].

## Methods

The cross-sectional MATCH study was designed to compare client experiences of different HIV testing services in Burkina Faso, Kenya, Malawi and Uganda. It was conducted in 2008-09, after ART programs had been scaled up in all four countries. The MATCH study’s methods have been reported in detail elsewhere [19]. In all four countries, HIV counselling and treatment (HCT) policies emphasize partner disclosure.

The MATCH study was based on a main survey (including open-ended questions on disclosure and its consequences) of clients at health facilities offering HCT in the capital region and one rural province/district in each country. Health facilities were purposefully selected and consisted of: (1) integrated facilities (hospitals and medical facilities providing both provider- and client-initiated testing alongside medical services); (2) antenatal clinics and other facilities offering care to pregnant women; and (3) standalone facilities for HCT.

Potential participants were randomly approached at the study sites. After receiving their informed consent, field interviewers administered face-to-face questionnaires including multiple choice and open-ended questions (n = 3,659). The collected data included respondents’ socio-demographic characteristics, reported HIV status, time elapsed since learning their status, their experiences with HIV counselling and testing and partner disclosure, as well as reported experiences of self- and enacted stigma. To triangulate the information collected through the survey’s open-ended questions, we conducted 102 in-depth interviews with key informants recruited through support groups for HIV-positive individuals. To gain further insight into the lives of HIV-positive persons, experienced qualitative researchers conducted 20 focus group discussions with support group members.

### Qualitative analysis

To assess the dynamics and consequences of partner disclosure and to develop hypotheses on its determinants, we analysed the partner disclosure narratives of 157 HIV-positive individuals contained in the responses to the survey’s open-ended questions (see Table 1 for their demographic characteristics). For each respondent, we wrote a case summary citing their reasons for being tested, whether health workers had encouraged them to disclose, their reasons to disclose to their partner, reactions to disclosure from their partner, their experiences of self-stigma, and whether they had joined a support group. Insights into the dynamics of disclosure were also derived from in-depth interviews and focus group discussions in all four countries, which were analysed with NVivo 8 software.

**Table 1:** Demographic characteristics of missing records compared to the original sample

**Variable**		**All (n = 281)**	**Records excluded (n = 50)**
**Gander, Female: Male, n (%)**		174 (68.1): 107 (31.9)	27 (54): 23 (46)
**Age, mean (SD)**		34.5 (9.1)	35.1 (9.6)
**Education, <primary: > = primary, n (%)**		146 (51.9): 135 (48.1)	24 (48): 26 (52)
**Country**	**Malawi, n (%)**	114 (40.6)	13 (26.0)
	**Kenya, n (%)**	74 (26.3)	11 (22.0)
	**Uganda, n (%)**	51 (18.2)	19 (38.0)
	**Burkina Faso, n (%)**	42 (14.9)	7 (14.0)

### Quantitative analysis

Our quantitative analysis focused on the subset of MATCH participants who reported being tested, who were aware of being HIV-positive for more than one week, and who identified themselves as married or cohabiting. The dependent variable was self-reported disclosure to partners.

**Figure 1:**
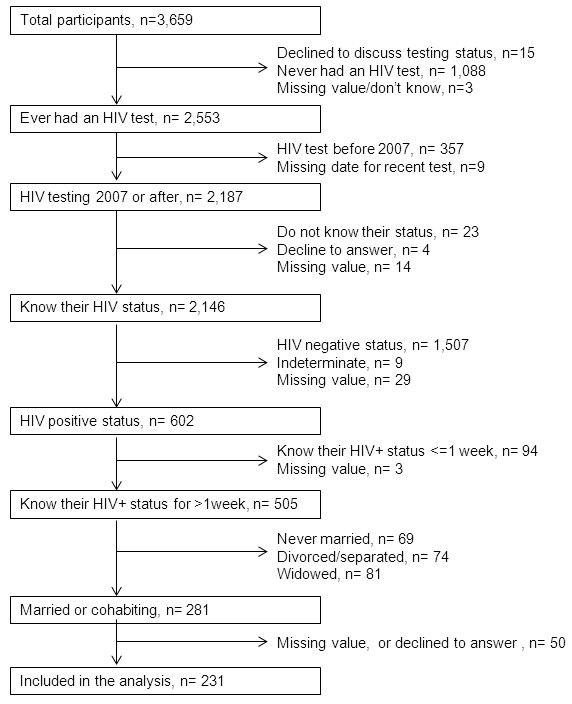
**Participant selection.** This figure shows the selection process of participants. From the total sample of 3,659, we excluded people in the following order: (1) participants lacking information on their testing status or who were never tested; (2) participants who tested before 2007; (3) participants lacking information on their sero-status; (4) participants who were HIV-negative or were unwilling to discuss their sero-status; (5) participants who knew their sero-status for less than one week; (6) participants not currently married and/or cohabiting; (7) participants lacking information or not willing to discuss disclosure to their partners. n, refers to number of participants; missing value/don't know refers to participants who did not answer the relevant question or answer not being aware of that information; >1 week, duration more than one week.

Of the total sample, 69.8% of respondents (n = 2,553/3,659) responded affirmatively to the question whether they had ever had an HIV test. Of those who had tested in or after 2007 and knew their HIV status, 28.1% (n = 602/2,146) reported being HIV-positive; 83.9% of this latter group (n = 505/602) had been aware of their sero-status for more than one week. A further subset of this group (55.6%, n = 281/505) identified themselves as married or cohabiting (Figure 1). After excluding 50 (17.8%) individuals with missing data on disclosure to their partners, our sample included 231 individuals. We provide a description of demographic data of missing records in Table 1.

The determinants of partner disclosure to be tested were defined by the results of the analysis of the open-ended answers of 90 HIV-positive women and 67 HIV-positive men (see above section on qualitative analysis). These included: (1) individual characteristics (gender, age, education, recruited in a rural or urban area, and the presence of symptoms requiring HIV testing or treatment at one’s most recent test); and (2) membership in support groups and (3) experiences of stigma. All were included as categorical variables, except for age which was included as a continuous variable.

We did not include measures of enacted stigma in our quantitative analysis as our qualitative findings suggested that it could be both a determinant of non-disclosure and a consequence of disclosure. Understanding the relationship between enacted stigma and disclosure would require a longitudinal study. We did, however, examine (fear of) enacted stigma in our qualitative analysis.

We expected self-stigma to affect disclosure – as had been reported in other studies [12,13,31] – and included self-stigma (inner feelings of worthlessness and/or guilt) in our quantitative analysis. We created a dichotomous variable for reported self-stigma, based on a positive response to either of two questions: “I sometimes feel worthless because I am HIV-positive” and “I feel guilty because I have HIV”. We derived this definition of self-stigma from the World Health Organization’s manual *HIV Testing, Treatment and Prevention: Generic Tools for Operational Research*[32].

Membership of support groups was self-reported. We ran tests of bivariate associations and modified Poisson regressions to assess significance and to estimate the unadjusted and adjusted associations between selected determinants and the dependent variable [33]. The first multivariate model included each determinant and country of recruitment. A second multivariate model included all variables significant at p-value < 0.05 in the univariate analysis and country of recruitment. We analysed the data using Stata/SE 11.2.

The study was approved by the Ethics Review Committee of the World Health Organization, and by an Institutional Review Board (IRB) in each of the four countries (the Comité d’Ethique pour la Recherche en Santé of the Ministries of Health and Higher Education in Burkina Faso; the Institutional Review Board of Kenyatta National Hospital in Kenya; the National Health Science Research Committee of the Ministry of Health and Population in Malawi; and the Institutional Review Board of the National Council for Science and Technology of Makerere University in Uganda).

## Results and discussion

The analysis of participant (90 female and 67 male) responses to the survey’s open-ended questions pointed to three main reasons for disclosing in all four countries: (1) prevention, including the intention to use condoms and/or encourage one’s partner to get tested (reported more often by women); (2) to receive care and support from one’s partner (reported more often by men); and (3) the intimate nature of the relationship (mentioned by both men and women).

The logic of prevention informed the decision to disclose for many respondents. A 33 year-old Malawian woman diagnosed in antenatal care said she disclosed after being advised to do so by healthcare workers; she wanted her husband to be tested as well. She had expected to receive a positive diagnosis since her husband had multiple wives. She experienced stigma in the past when she was told to eat alone. She joined a support group. A 28 year-old Malawian woman tested at a VCT centre said she disclosed to her husband because she wanted him to accept using condoms. Her husband had encouraged her to get tested. Referring to her husband’s reaction, she says, “*He was happy that I made the decision to get tested, we are both on ART and living happily”*. She has experienced stigma when people were laughing at her. A 51 year-old man in Burkina Faso tested in a standalone VCT facility in urban Dédougou, because he had lived with a girlfriend who had tested positive and had subsequently died. Health workers had not encouraged him to disclose to his current wife. Before he received his results he told his wife to get tested, while she tested negative his results were positive. He decided to disclose to his new wife to protect her: *“I could not hide it from her. It is to save her, that’s why I told her”.*

Some respondents disclosed to their partners so that they could receive the care they needed. A 58 year-old married man in Uganda was routinely tested for HIV when he was admitted in a rural hospital for TB. It was difficult for him to be tested; *“I thought that when I was told my HIV results I would die immediately”.* He was not encouraged by health workers to disclose. While he feared disclosing because he feared that his wife would leave him he decided to disclose to his partner and brother *“to get support and care from them”*.

Others referred to the intimacy of their relationships. A 37 year-old woman from Burkina Faso had tested initially since she was suffering from regular bouts of malaria. She was not encouraged by a health worker to disclose but said she wanted to tell her husband because *“although we have no children we have been together for two years”*. In the absence of children, who are an important element for a woman’s social standing, this woman valued the relatedness to her husband. There were varied ways how the respondents describe the intimacy of their relationship, some simply commented that they disclosed *“because we are living together”*. Others stated that their partners *“deserved to know the truth”* such as a married 43 year old Ugandan man who tested at a standalone VCT center in rural Soroti. He did not discuss going for a test with his wife beforehand. The health workers told him not to be ashamed and encouraged him to disclose. He disclosed to his wife; *“It’s because her being my wife I had to tell her the truth”.*

### Consequences of disclosure

The analysis of disclosure narratives revealed that acceptance of a partner’s HIV status is a gradual process. While the initial reaction was often one of shock and disbelief, most partners grew more supportive over time. A 27 year-old married woman from Kenya explained that she was tested for HIV when she was pregnant. Her husband at first did not believe the result: *“He was shocked and could not believe it. We both went to test together”.* After he was tested, he became more supportive and encouraging. A 28 year-old married woman from Uganda was admitted with fever to the Mulago TB ward where she was encouraged to test for HIV. She could not believe that she was HIV-positive because she had been faithful to her partner. Although health workers did not encourage her to disclose, she decided to tell her parents and husband *“so that he could also go and test so that we can plan and look after our children”*. Though his initial reaction was discouraging, it improved over time: *“He felt bad, condemned me that I was the one who brought the infection. [But later] he gave me money for treatment and transport”.*

The 157 partner disclosure narratives revealed that partners’ initial reactions can vary tremendously. Negative responses ranged from disbelief and denial, blaming the spouse for infidelity and bringing the disease into the family, to violence, separation and divorce. In some cases there was a complete absence of reaction, interpreted as indifference. But when respondents described supportive responses, they described their partners as *“understanding”* and *“loving”*.

Reactions to disclosure differed by gender. 24 out of 90 women reported receiving negative reactions, which was the case for only 7 out of 67 men (four in Kenya, two in Uganda, and one in Malawi). A 37 year-old woman in Burkina Faso chose to disclose to her husband because she wanted him to get tested. When she told him, he shook his head, left her with their children, and cut off his support. When his new wife arrived, the respondent had to move in with her uncle. She reported that her ex-husband had told his new wife that she had *“walked with other men”*, which had given her the disease, but that he was healthy.

One of the Kenyan men who suffered a negative response from his partner was a 34 year-old who tested for HIV when he was being treated for a sexually transmitted infection. Health workers encouraged him to disclose to his wife, with whom he had been living for five years. When he did so, she accused him of infidelity, saying he had *“gotten infected elsewhere”*. They have since divorced. The divorce, alongside knowing that he *“cannot marry again”*, has been the most difficult thing so far about living with HIV.

### Reasons for non-disclosure

In explaining their decisions not to disclose, most respondents alluded to the fear of enacted stigma. A 38 year-old man from Malawi, was tested in Mitundu Rural Hospital because he wanted to know his status since he was falling sick often, he discussed going for a test with his wife beforehand but he did not disclose his results because he wanted *“to avoid social abuse”*. A 27 year-old woman in Burkina Faso who went to a VCT centre to get tested because of her husband’s sexual behaviour reasoned similarly: “*If you tell them that you have the disease, they will reveal your name. If you have a co-wife and she learns that you have the disease, it will cause you harm”.* In Uganda, a 19 year-old woman who was routinely tested for HIV when she was pregnant had known her status for two and a half months at the time of the interview and did not disclose because *“My husband would divorce me even if he were to know that he is infected”.*

Interestingly, three men in Burkina Faso reported that they had been advised at three different testing facilities (two at VCT centres and one at an integrated health facility) to not disclose their test results. A 68 year-old man who had tested at a standalone VCT facility in urban Dédougou stated that he had not disclosed *“because we were given advice there that everyone has to keep his findings secret. That’s why I have not spoken to anyone, even my wife”.*

### The role of support groups

Support groups can provide HIV-positive individuals with information on how to disclose to their partners. Our interviews with key informants revealed that support groups can also counsel confidentiality. Many support groups seem to advocate selective and timely disclosure. Members of support groups whom we interviewed reported:

In the support group meetings, we have been told that you don’t have to disclose if you are not ready. But it is good to disclose if you want to (woman, mid-40s, Kenya).

I learnt from the support group that disclosure is a process. It is not a one-time event. If I had known this before, I would not have disclosed to my wife so soon. If I had done it more gradually, the impact would not have been that bad. If you don’t take time to disclose, and just tell someone that you are positive, like I did with my wife, the person will think that you are about to die. He will even think that sharing food with you will infect them with HIV (man, 44 years, Kenya).

I have not told anyone yet. The only people who know are the support group members. I have not told my children but they know that I take medicine daily. I have not told anyone because if you tell people they will just stigmatize you (woman, 30 years, Kenya).

Male support group members in a focus group in urban Malawi argued that their membership was already a form of disclosure. As one respondent explained:

*I didn’t disclose to anybody because I don’t know their status, why should I trouble myself in disclosing mine to people who would not respect me? But because I am a member of a support group here at least they know my sero-status*.

#### Quantitative results

We used our qualitative findings to develop hypotheses on the determinants of disclosure. The following section describes our quantitative analysis of these hypotheses.

As shown in Table 2, the majority of participants in our sample (N = 231) were women (63.6%, n = 147/231). The average age was 34.4 years. Almost half (47.2%, n = 109/231) reported having finished at least primary education. The majority were recruited in urban or peri-urban settings (58.1%, n = 133/229). The overall rate of reported self-stigma was 23.1% (n = 52/227). Almost three-quarters of our respondents did not report any symptoms requiring testing or treatment for HIV at the time of their last test (71.5%, n = 163/228). 60.6% of respondents [95% CI 54.0-67.0] (n = 140/231) reported disclosing their sero-status to their partners; all reported disclosing to someone.

**Table 2:** Sample (n = 231)

**Variable**		**N (%)**	**Disclosure, n (%)**
**All**		231 (100)	140 (60.6)
**Gender**	female	147 (63.6)	77 (52.4)
	male	84 (36.4)	63 (75.0)
**Age**	mean (SD)	34.4 (9.0)	35.3 (9.2)**
**Education**	<primary	122 (52.8)	60 (49.2)
	> = primary	109 (47.2)	80 (73.4)
**Setting of recruitment**	rural	96 (41.9)	39 (40.6)
	urban	133 (58.1)	113 (72.9)
Presence of symptoms requiring treatment at most recent test*	no	163 (71.5)	106 (65.0)
	yes	65 (28.5)	32 (49.2)
Self-stigma*	no	173 (76.9)	103 (59.5)
	yes	52 (23.1)	37 (71.2)
Membership in support groups*	no	155 (68.9)	113 (72.9)
	yes	70 (31.1)	27 (38.6)
**Country of recruitment**	Malawi	101 (43.7)	33 (32.7)
	Kenya	55 (23.8)	51 (92.7)
	Uganda	40 (17.3)	31 (77.5)
	Burkina Faso	35 (15.2)	25 (71.4)

**Legend:***N* = number of participants in each group; *n* = number of participants reporting disclosure to their partners; *SD* = standard deviation; <primary: incomplete primary education or no education; > = primary: complete primary education and above. *Values do not add up to overall total due to missing values. **mean and standard deviation of those disclosing to partners.

Partner disclosure levels in Kenya, Uganda and Burkina Faso were much higher at 92.7% (n = 51/55, p < 0.001), 77.5% (n = 31/40, p < 0.001) and 71.4% (n = 25/35, p < 0.001) respectively than in Malawi, at only 32.7% (n = 33/101).

### Determinants of disclosure

Table 3 shows the determinants of disclosure to partners. In univariate analysis, men were more likely to disclose (uRR 1.43 [95% CI 1.18-1.75], p < 0.001), as were older respondents (uRR 1.01 [95% CI 1.00-1.02], p = 0.042). Respondents recruited in urban settings were more likely to disclose (uRR 1.85 [95% CI 1.43-2.40], p < 0.001) than those recruited in rural settings.

**Table 3:** Determinants of disclosure to partners

**Variable**		**uRR [95% CI]**	**p value**	**aRR [95% CI]**^**1**^	**p value**	**aRR [95% CI]**^**2**^	**p value**
**Gender**	female	**1**		**1**		1	
	male	**1.43 [1.18-1.75]**	**<0.001**	**1.22 [1.02-1.46]**	**0.026**	1.06 [0.87-1.31]	0.563
**Age**		**1.01 [1.00-1.02]**	**0.042**	**1.01 [1.00-1.02]**	**0.017**	**1.01 [1.00-1.02]**	**0.030**
**Education**	<primary	**1**		1		1	
	> = primary	**1.49 [1.21-1.85]**	**<0.001**	1.12 [0.90-1.40]	0.305	1.02 [0.81-1.29]	0.842
**Setting of recruitment**	rural	**1**		1		1	
	urban	**1.85 [1.43-2.40]**	**<0.001**	1.28 [0.99-1.64]	0.056	1.23 [0.96-1.59]	0.107
**Presence of symptoms requiring treatment**	no	**1**		1		1	
	yes	**0.76 [0.58-0.99]**	**0.045**	0.81 [0.62-1.06]	0.128	0.75 [0.58-0.98]	0.032
**Self-stigma**	no	1		1		1	
	yes	1.20 [0.97-1.48]	0.101	0.94 [0.79-1.11]	0.454	n/a	n/a
**Membership in support groups**	no	**1**		**1**		1	
	yes	**0.52 [0.39-0.72]**	**<0.001**	**0.65 [0.49-0.86]**	**0.003**	**0.67 [0.51-0.88]**	**0.004**
**Country of recruitment**	Malawi	1		n/a	n/a	1	
	Kenya	**2.84 [2.12-3.79]**	**<0.001**	n/a	n/a	**2.05 [1.49-2.82]**	**<0.001**
	Uganda	**2.37 [1.71-3.29]**	**<0.001**	n/a	n/a	**1.74 [1.21-2.50]**	**0.003**
	Burkina Faso	**2.19 [1.54-3.10]**	**<0.001**	n/a	n/a	**1.89 [1.29-2.75]**	**0.001**

**Legend:** uRR [95% CI]: unadjusted risk ratio and 95% confidence interval; aRR [95% CI]: adjusted risk ratio and 95% confidence interval; <primary: incomplete primary education or no education; > = primary: complete primary education and above; n/a: not applicable. Adjusted RR are presented: ^1^ adjusted for country of recruitment only; ^2^ multivariate model including gender, age, membership in support groups, and country of recruitment.

Membership in support groups was strongly associated with *not* disclosing to partners (uRR 0.53 [95% CI 0.39-0.72], p < 0.001). Having symptoms requiring further diagnosis or treatment at one’s most recent test was also associated with non-disclosure to partners, though the association is less strong (uRR 0.76 [95% CI 0.58-0.99], p < 0.001).

In multivariate analysis, country of recruitment remained associated with disclosure rates. Participants from Kenya, Burkina Faso and Uganda reported significantly higher rates of disclosure to partners than in Malawi. Membership in support groups, presence of symptoms requiring treatment, and age remained associated with disclosure (aRR 0.67 [95% CI 0.51-0.88], p = 0.004; aRR 0.75 [95% CI 0.58-0.98], p = 0.032; and aRR 1.01 [95% CI 1.00-1.02], p = 0.030 respectively).

## Discussion

Our findings show that partner disclosure rates in times of treatment scale-up are high in Kenya, Uganda and Burkina Faso, where the majority (respectively 92.7%, 77.5% and 71.4%) of respondents reported having disclosed their HIV-positive status to their partners. The disclosure rate is much lower in Malawi, where only one-third of our respondents reported having disclosed to their partners. Disclosure was positively associated in univarite analysis with being older, living in urban areas, higher education levels, and being male, while being negatively associated with membership to support groups and presence of symptoms requiring treatment. The multivariate analysis shows age, presence of symptoms requiring treatment, membership to support groups and country of recruitment to be independently associated to the prevalence of disclosure.

Analysis of the disclosure stories revealed varying reasons to disclose to partners in all four countries. In some cases, HIV-positive individuals wanted to protect their partners from infection (the reason mentioned most by women). In other cases, healthcare needs drove the disclosure process (mentioned more often by men). Both men and women also referred to upholding the integrity of their relationship as a reason to disclose to their partner. The differences by gender in reasons to disclose may be related to the fact that men more often test late, when they are already sick. Women are tested more often in PMTCT, when they are not yet sick.

One in four survey respondents scored positively on our measures for self-stigma. But in our multivariate analysis, self-stigma was not associated with disclosure. Self-stigma was rarely referred to in the open-ended responses. However, the disclosure narratives suggest that *fear of enacted stigma* is an important reason for non-disclosure for both men and women. Given the often negative reactions HIV-positive individuals report when they disclose to their partners, the fear of enacted stigma seems justified. Women reported more negative consequences than men, though the stories also revealed changes in response where negative reactions from partners became more supportive over time.

These findings have implications for PMTCT programs which involve routine HIV testing and early treatment initiation in antenatal care. Health workers routinely encourage HIV-positive pregnant women to refer their partners for testing, though in reality this seems easier said than done. As we have argued elsewhere, home-based testing may be a more gender-neutral way of scaling up HIV testing, as both men and women can be tested simultaneously at home [26].

Our interviews and focus group discussions with members of support groups suggested that support groups advocate caution when disclosing to partners. HIV-positives should disclose when they are ‘ready’ to face the consequences. In light of the negative partner responses reported by many respondents, this makes sense. A key finding of our multivariate analysis is that membership in support groups is associated with lower levels of disclosure to partners. This finding is independent of the country of recruitment, gender, and education.

The current analysis has certain limitations. Its qualitative findings cannot be generalized. Small numbers of HIV positive who answered the open ended questions made it impossible to analyse subtle differences in disclosure dynamics by country. The gender dynamics that we reported need to be further analysed in larger scale studies that can account for the differential effects of HCT practices on partner disclosure. Women who test in PMTCT are given different advice than men who test in provider initiated testing and counselling (PITC) programs in general health clinics. How do these different health care constellations affect the observed differences in motivations to disclose?

The role of support groups in disclosure processes needs to be further examined. Is the association observed between support group membership and non-disclosure caused by a tendency of people who fear enacted stigma to attend these groups? A study in five African countries found that individuals participating in regular support groups reported experiencing greater stigma (the study treated enacted and feared stigma as one). The study hypothesized experienced stigma as a reason why people participate in support groups in the first place [34].

Finally, our mixed-methods study was unable to explain the differences observed in partner disclosure rates between countries due to our inability to stratify the analysis (small sample size). Why are they lower in Malawi than in the other three countries? We suspect that differences in HTC – both in policy and in how services are delivered – might be important part of the story. Counsellors in Malawi possibly place less emphasis on partner disclosure, or gender relations may be different in the country. Future mixed-methods studies should combine measurement of disclosure rates with observation of counselling practices on the ground, with more attention for gender relations and kinship dynamics.

We also recommend that future studies follow longitudinal designs to better grasp the dynamics that affect disclosure to partners. New models are needed to understand how (fear of) enacted stigma is associated with, becoming members of support groups and partner disclosure. Our qualitative findings reveal that women often face negative responses when they disclose and that their fear of enacted stigma might be justified, in their individual stories. Further questions include - what role do support groups play? And how they protect people from the risks of enacted stigma?

## Conclusion

Disclosure dynamics are context-specific. They are influenced by the practices of healthcare providers and support groups, by cultural views on HIV/AIDS as well as by kinship dynamics and gender relations. Qualitative studies are needed to better understand how contextual factors affect partner disclosure.

Understanding the dynamics of partner disclosure has become crucial now that Treatment as Prevention programs (involving the early initiation of ART) are being scaled-up in Sub-Saharan African settings for sero-discordant couples. As disclosure tends to be motivated by both the need for support for oneself as well as caring about the partner’s well-being, the message that treatment has benefits for both partners is worth emphasizing. Support groups may prove suitable venues for discussing the benefits of treatment not only for one’s own health but also one’s sexual partner. Their role in supporting people to initiate early treatment and disclose to partners should be stressed more in scaling up HIV testing and antiretroviral treatment.

We have pointed to the prevailing gender dynamics in access to HIV testing. Women tend to be tested early in PMTCT programs, while men come late for testing when they are already ill. As a result women tend to be burdened with the need to disclose. Elsewhere we have argued for home-based testing to overcome this gender inequality in access to HIV testing and disclosure burden [26,35]. Home-based testing enables simultaneous testing of sexual partners in the privacy of their homes, creating an opportunity for post-test couple counselling in which the benefits of treatment for both the HIV positive and the HIV negative partner can be emphasized.

## Abbreviations

ART: Antiretroviral treatment; HIV: Human immunodeficiency virus; IRB: Institutional review board; PITC: Provider initiated testing and counselling; PMTCT: Prevention of mother-to-child transmission; VCT: Voluntary counselling and testing; WHO: World Health Organization.

## Competing interests

This project was supported by a grant from the National Institutes of Health (5 R01 HD053268-05), PI: Carla Obermeyer (CO). This support is gratefully acknowledged. The National Institutes of Health had no role in the study design, data collection and analysis, decision to publish, or preparation of the manuscript. At the time of the study, CO was a scientist at the World Health Organization (WHO), and the support of WHO is gratefully acknowledged. The WHO did not define the project or influence data collection or interpretation.

## Authors’ contributions

Concept protocol. This analysis is part of a larger research project, the proposal of which was developed by CO. AH designed the analysis of the subset of data on which this paper is based.

Data collection. CO and AH jointly developed the quantitative and qualitative research instruments in consultation with researchers (AD, RW, OK, IN, EK, JK) from the country teams. Data collection was done by the country researchers (RW, OK, IN, EK) together with research assistants.

GG conducted the quantitative analysis with input from AH, EV, MN and EM. AH, EV and CS conducted the qualitative analysis. AH and EV were responsible for the triangulation of results, where some quantitative findings were nuanced with qualitative insights. The conclusions were validated by the country researchers (RW, OK, IN, EK).

Manuscript draft. The manuscript was written by AH, GG and EV. All authors commented on the first draft of the paper. Comments were incorporated in the final draft, which was sent around for approval before submitting. All authors read and approved the final manuscript.

## Pre-publication history

The pre-publication history for this paper can be accessed here:

http://www.biomedcentral.com/1471-2458/13/589/prepub
